# Genetic Risk Scores for Diabetes Diagnosis and Precision Medicine

**DOI:** 10.1210/er.2019-00088

**Published:** 2019-07-19

**Authors:** Miriam S Udler, Mark I McCarthy, Jose C Florez, Anubha Mahajan

**Affiliations:** 1 Diabetes Unit, Massachusetts General Hospital, Boston, Massachusetts; 2 Center for Genomic Medicine, Massachusetts General Hospital, Boston, Massachusetts; 3 Programs in Metabolism and Medical and Population Genetics, Broad Institute of MIT and Harvard, Cambridge, Massachusetts; 4 Department of Medicine, Harvard Medical School, Boston, Massachusetts; 5 Oxford Centre for Diabetes, Endocrinology and Metabolism, University of Oxford, Headington, Oxford, United Kingdom; 6 Wellcome Centre for Human Genetics, University of Oxford, Oxford, United Kingdom; 7 Oxford NIHR Biomedical Research Centre, Oxford University Hospitals NHS Foundation Trust, John Radcliffe Hospital, Oxford, United Kingdom

## Abstract

During the last decade, there have been substantial advances in the identification and characterization of DNA sequence variants associated with individual predisposition to type 1 and type 2 diabetes. As well as providing insights into the molecular, cellular, and physiological mechanisms involved in disease pathogenesis, these risk variants, when combined into a polygenic score, capture information on individual patterns of disease predisposition that have the potential to influence clinical management. In this review, we describe the various opportunities that polygenic scores provide: to predict diabetes risk, to support differential diagnosis, and to understand phenotypic and clinical heterogeneity. We also describe the challenges that will need to be overcome if this potential is to be fully realized.

**Essential Points**

- During the last decade, there have been major advances in our understanding of the genetic basis of the most common subtypes of type 1 (T1D) and type 2 diabetes (T2D), with >500 robust associations identified
- Although individual variants typically have only a modest effect on risk, when combined into a polygenic score, they offer increasing power to capture information on individual patterns of disease predisposition with the potential to influence clinical management
- The generation of polygenic scores based on overall T2D predisposition can identify individuals with a high future risk of diabetes who may benefit from targeted interventions
- The generation of polygenic scores based on overall T1D risk can identify individuals who may benefit from early interventions to forestall the risk of T1D, and it also supports the identification of those with later-onset diabetes who have an autoimmune etiology, for whom early recourse to insulin therapy may be advantageous
- The generation of partitioned polygenic scores that capture aspects of the etiological and clinical heterogeneity that contributes to variable clinical outcomes in those with T2D has the potential to deliver clinical benefit through enhanced capacity to predict disease progression, complication risk, and response to pharmacological and behavioral interventions
- Polygenic scores have predominantly been derived from genetic studies performed in European populations and have suboptimal ability to capture risk in individuals of non-European origin
- Although there are a number of technical and logistical issues to be addressed before the clinical utility of polygenic scores can be fully enumerated, increasing utilization of polygenic scores within diabetes clinical practice is likely to be an important component of efforts to deliver precision medicine for those who have, or are at risk for, diabetes

Diabetes is already one of the major contributors to death and ill health globally, and its prevalence continues to rise. Current projections estimate almost 500 million affected by diabetes as of 2017 (and almost 700 million by 2045), most of this in the form of type 2 diabetes (T2D) (1). Escalating rates of T2D speak to the limits of current strategies for prevention, whether they involve lifestyle interventions (for example through dietary modification and increased physical activity) or pharmacotherapy. At the same time, the burden of disease arising from the complications of inadequately controlled diabetes (manifested as renal failure, vision loss, amputation, and accelerated vascular disease) highlights the urgent need for major improvements regarding both the timely diagnosis of diabetes (because much damage is initiated while the disease is subclinical) and the management of those with established disease.

The condition that we currently label as “type 2 diabetes” represents a convenient, but likely suboptimal, construct for the application of 21st century medicine. Although individuals with established T2D have a generalized metabolic derangement (typically associated with hyperlipidemia, adiposity, disturbed hepatic metabolism, and the like), formal diagnosis rests entirely upon a single metabolic component—glucose—itself the end result of multiple metabolic processes. The diagnosis of diabetes depends on numeric thresholds placed within continuous distributions of (fasting, random, or postprandial) glucose and/or glycated Hb levels. These thresholds were initially based around the observed relationships between levels of hyperglycemia and the incidence of specific diabetic complications, such as retinopathy, but they may not be equally discriminating for the macrovascular complications (2). Crucially, T2D remains effectively a diagnosis of exclusion, made after those with hyperglycemia attributable to more defined causation, including islet autoimmunity [type 1 diabetes (T1D)], highly penetrant genetic effects [*e.g.*, maturity onset diabetes of the young (MODY)], and certain specified exposures (steroids, pancreatitis, pregnancy), have been excluded. Those left with the diagnosis of T2D demonstrate considerable heterogeneity with respect to presentation, clinical course, and response to available therapies, yet clinical pathways tend to be based around universally applied algorithms that take little, if any, account of that heterogeneity (3–5).

Human genetics provides a powerful set of approaches for addressing some of these challenges, delivering both an improved understanding of the mechanisms contributing to the development of diabetes and opportunities for direct translational benefit (6). Both common major subtypes of diabetes (T1D, T2D) are complex, multifactorial traits; that is, an individual’s risk of developing either of these conditions is influenced by the combination of genetic variation at multiple sites across the genome, acting in concert with factors within the external (*e.g.*, nutritional availability, socioeconomic status) and internal (*e.g.*, microbiome, metabolic memory) environment (7, 8). During the last decade, large-scale genetic studies [typically in the form of genome-wide association studies (GWAS)] have identified >400 distinct genetic signals influencing T2D risk (9) and >50 with impact on T1D predisposition (10). Most of these DNA sequence variants are widely shared within and between populations, in contrast to the more private alleles that drive some rarer subtypes of diabetes (11, 12). With the notable exception of the HLA region (which has the major impact on T1D risk), most of these common variants have only modest effects on individual predisposition: the biggest effects for T2D modulate risk by no more than 40% per allele, and most have much smaller effects (9, 10).

However, in combination, the impact of this variation can be more profound (9, 13). In the most recent GWAS for T2D, the entire set of associated variants so far detected explains ∼20% of the overall variation in disease risk (9), in Europeans at least (comparable analyses in non-European populations are limited by the sample sizes available for study). Estimates of the heritability of T2D vary widely (14, 15) around a median of 40%, suggesting that around half the genetic contribution to the variation in risk can be quantified for each individual. Estimates of the heritability of T1D are higher (16), and a greater proportion of that genetic risk can be captured using existing approaches. Ongoing efforts to further characterize the genetic basis of both major subtypes of diabetes—through detecting significant associations at variants that have escaped detection because they are too rare or have small effects—will increase the proportion of individual genetic predisposition that can be directly measured.

The steadily expanding list of genetic variants delivered by these successive waves of genetic discovery has delivered novel mechanistic insights into disease pathophysiology. Some of these insights have led to an understanding of the major processes contributing to disease risk, such as the role of islet-specific as well as immunological processes with respect to T1D risk (17, 18) or the relative impact of defects in insulin secretion and action for T2D (19). Other studies have attempted to dissect the detailed molecular, genomic, and physiological events that mediate risk at individual loci (9, 20–23). These efforts can have direct translational impact, for example through the identification of novel therapeutic targets, or biomarkers that track disease progression.

In this review, however, we focus on a different route from human genetics to translation, one that derives estimates of an individual’s predisposition to diabetes and its subtypes (in the form of polygenic scores) from the patterns of individual geneticvariation at sites known to influence diabetes predisposition.

## The Concept of Polygenic Scores

The idea of grouping genetic variants to capture the aggregate genetic risk for a given disease is not new. An early promise of genetic discovery in complex (polygenic) conditions was to predict clinical outcomes. It was recognized that, in contrast to classical Mendelian diseases, where the presence of a specific mutation was deterministic and typically heralded the eventual onset of disease (contingent on penetrance), the genetic risk for complex, multifactorial diseases is probabilistic and most appropriately used as a predictor that quantified a discrete increment in overall risk (24). This is because for complex human traits, the overwhelming majority of associated genetic variants exert modest effects, and the ability of any individual variant to influence clinical outcomes is small. The obvious approach is to sum the effects of risk alleles associated with a given condition, to generate an aggregate estimate of genetic risk. This approach was justified by the observation that early genetic associations seemed to work in an additive fashion, with little or no evidence of epistasis. This concept was pioneered for age-related macular degeneration, the first disease for which GWAS proved successful (25) and had also been used in T2D for the three reproducible genetic associations that had emerged from the pre-GWAS era (26).

This concept can be easily expanded from the disease arena to quantitative traits. Here, rather than expressing “risk” (which connotes the deleterious burden of illness), the aim is to capture the overall variance in a trait conferred by the set of genetic variants grouped into a composite score. Examples include circulating levels of a specific metabolite or the inherited predisposition toward a behavioral pattern, where the deleterious connotations ascribed to the term “risk” no longer apply. In this review, therefore, we favor the use of the term “polygenic score” as a more inclusive general descriptor.

The initial uses of polygenic scores deliberately focused on the inclusion of individual genetic variants for which the evidence for association was robust. This occurred as a “route correction” to the historical trend whereby the proliferation of candidate gene studies and the adoption of liberal statistical significance thresholds had led to the publication of many genetic associations that later proved irreproducible, and likely represented false-positive findings (27). In the GWAS era, such high-likelihood variants had to achieve genome-wide significance, based on a widely accepted threshold of *P* < 5 × 10^−8^, established to account for the estimated 1 million independent tests that exist among common variants in the European genome (28). Thus, polygenic scores began to be constructed through the compilation of genome-wide significant variants emerging from successive and ever-larger GWAS, each with increased statistical power (29).

**Figure 1.**
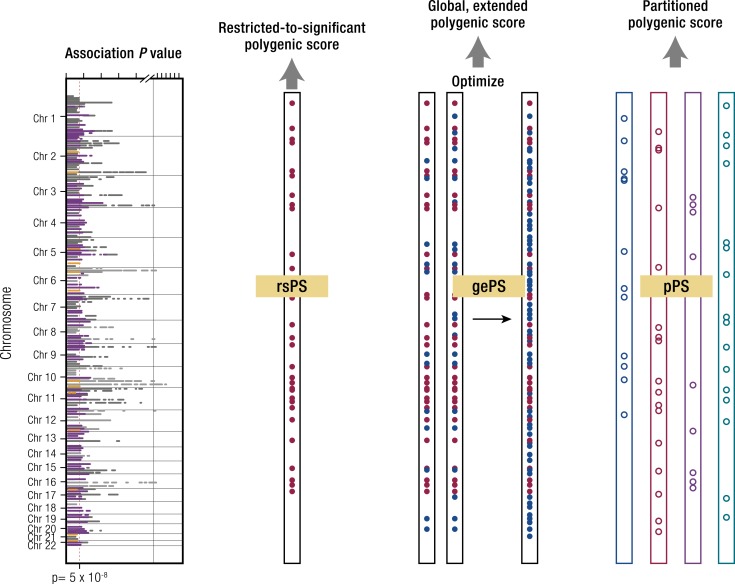
How polygenic scores are derived. The orange dashed line in the graph represents the threshold for genome-wide significance in a GWAS study. The filled red dots in the rsPS and gePS sections represent genetic variants reaching genome-wide significance, and the filled blue dots variants that have not reached genome-wide significance. In the pPS section, open dots reflect variants that have been assigned to one of the four groups of partitioned loci. For full explanation see text.

In the literature to date, scores that incorporate only variants that are individually significant (typically weighted to reflect their respective effect sizes on the trait of interest) have often been described as “genetic [risk] scores,” sometimes in contrast to use of the term “polygenic scores” to reflect those that build in additional subsignificant variants. However, these terms have been applied inconsistently and sometimes interchangeably, and in this review we take the opportunity to (re)define these concepts with labels that are easier to interpret. Because the former are composed of variants at the top or extreme of the statistical distribution, we propose the term “restricted-to-significant polygenic scores” (rsPS) (Fig. 1; see Appendix A).

In T2D, the use of rsPS was pioneered in a series of publications in 2008, each of which constructed an rsPS from the 16 to 18 T2D-risk variants known at the time (30–32) and compared its predictive performance to that of clinical T2D-risk factors. In Framingham samples, for instance, individuals with a “high” rsPS (score ≥21, ∼11% of the cohort) had 2.6 higher odds of developing T2D than did those with a “low” rsPS (score ≤15, ∼25% of the cohort) (31). We discuss these rsPS studies in detail later in this review.

Although compiling variants that achieve genome-wide significance ensures that the variants included in the score represent real associations with disease, such a stringent threshold ignores many other variants that, although truly associated with the phenotype, have escaped detection at genome-wide significance due to limited sample sizes. However, an estimate of their likely contribution is available in GWAS data sets, even when they fail to achieve genome-wide significance. Therefore, under the assumption that the effects of variants that have no association with disease will tend to cancel each other by random fluctuations around the null distribution, there is an opportunity to extend the polygenic score beyond the set of individually significant variants (including, potentially, all of the variants from the GWAS data set) in the expectation that the small cumulative effects of many hundreds or thousands of truly associated variants can contribute to the overall score, and improve power. In practice, the scores derived in this way do not usually include all variants. Typically, the full set of variants is pruned to selectively remove highly correlated variants: this pruning is combined with an optimization step that evaluates the discriminative performance of different sets of variants (defined using a range of progressively more liberal association *P* value cutoffs), to establish which cutoff maximizes the predictive signal (33). We propose the term “global extended polygenic scores” (gePS) to describe these extended polygenic scores. We prefer “global” over “total” in this context, because current approaches do not capture all aspects of genetic risk: private variants, many structural variants, and variants whose effect is modified by environmental factors are not optimally considered in these analyses.

The use of these global scores has been popularized recently with the assembly of large GWAS meta-analyses for multiple traits (13). Their increase in content allows for a steeper and more granular estimation of risk along the gradient of genetic burden. These scores can include many tens of thousands, even millions, of variants. For example, one such gePS for T2D risk, comprising 7 million variants, was able to demonstrate that, in the UK Biobank, individuals in the top 3.5% of a T2D gePS (generated from and optimized in a subset of independent UK Biobank samples) had an OR ≥3.0 when compared with the mean of the population (13).

The clinical manifestation of disease often reflects the confluence of multiple pathophysiological processes. In T2D, hyperglycemia typically requires the concomitant presence of insulin resistance and inadequate *β*-cell function. Each of these may in turn be caused by various mechanisms, such as incretin insufficiency and/or resistance, fatty acid accumulation, glucolipotoxicity, diet, inflammation, or the microbiome. To the extent that endophenotypes that reflect these processes can be captured in populations, one can estimate which of these processes is likely to mediate the T2D impact of each T2D-associated variant. Once these associations are established, discrete polygenic scores can be constructed with variants that share mediation of T2D risk through a specific intermediary process. For example, early efforts to group variants in this fashion revealed that one of the processes contributing to T2D risk involves insulin resistance that is characterized by lower levels of adiposity (34–36). This paradoxical combination of phenotypes, which reflects the pattern seen in a more extreme form in inherited lipodystrophies (37), likely reflects the consequences of an inherited defect in adipocyte development that limits the storage of excess lipid in “metabolically safe” fat depots.

One systematic approach to group variants in this way involves the use of clustering methods (described in more detail below). Here, investigators use orthogonal lines of evidence (*e.g.*, association with physiological measures of insulin secretion or resistance, pattern of expression of tagged genes, or open chromatin regions) to group genetic variants associated with T2D into specific clusters informed by biology (20, 38). We term these “partitioned” (or “process-specific”) polygenic scores (pPS) and explain below how these may help to define specific pathways that illuminate disease pathogenesis or highlight opportunities for pharmacological modulation. These partitioned scores may also, by capturing the endophenotypic profile driving an individual’s progression from health to disease, provide a framework for tailored preventive or therapeutic interventions.

It is worth emphasizing a critical point that is often neglected in the enthusiastic embrace of the burgeoning power of human genetics. Because, for complex traits such as T1D and T2D, inherited sequence variation is only one component of predisposition, even the best possible distillation of genetic potential will never provide a complete description of individual risk. A fuller assessment of present and future disease states for an individual requires the integration of genetic information with accurate and robust measures of other contributions to individual predisposition (including diet, lifestyle, and microbiome), as well as an assessment of current clinical state [including measurement of biomarkers such as glucose, lipids, islet autoantibodies, and clinical phenotypes such as body mass index (BMI) and waist-to-hip ratio]. The relative contributions of these various domains of information are likely to shift during life with measures of clinical state becoming ever more impactful in later life as disease becomes overt. However, as we will show, genetic variation has a critical part to play. The long-term stability of genetic variation, which is easily ascertained in peripheral blood, offers the potential for risk stratification throughout the life course; unlike other risk factors, it is also not subject to the confounding effects of disease or its treatment.

## Polygenic Scores in Action

### Predicting T2D onset

The slow onset of T2D, coupled to evidence that the damaging consequences often predate the clinical diagnosis by some years (2), emphasizes the clinical value of early diagnosis. The capacity for drugs and lifestyle interventions to lead to substantial reductions in the progression to diabetes (39, 40) motivates efforts to identify those at the greatest future risk of developing T2D. As discussed above, genetic predictors have the particular advantage of offering predictive information that is stable throughout life.

Prior to the first GWAS for T2D, three genetic variants had been associated with T2D with high confidence. Identified either through candidate gene analyses or the follow-up of linkage signals, these implicated *KCNJ11* p.E23K, *PPARG* p.P12A, and *TCF7L2* rs7903146. In 2006, Weedon *et al.* (26) assessed the combined risk of carrying these variants. As well as observing that the variants influenced T2D risk additively, the authors assessed the predictive value of the genetic tests using a standard approach that uses the tradeoff between the sensitivity and specificity of the test to generate a receiver operator characteristic (ROC) curve. The area under this curve (the AUROC, or C-statistic) provides a measure of the proportion of times such a test will correctly assign disease state between a pair of individuals, one who has the disease of interest (or, depending on the study design, would go on to develop it), and another who does not (or who remains disease-free on follow-up). The estimated AUROC was 0.58, exceeding the 0.50 value that indicates no discriminative capacity, but well short of the values seen for most clinically useful tests.

Publication of the first few rounds of T2D GWAS extended the number of significantly associated variants into the teens, enabling better powered studies (involving between 16 and 18 risk alleles) that sought to compare the value of an rsPS to predict incident diabetes to that of clinical factors alone (30–32). Lyssenko *et al.* (30) examined a 16–single-nucleotide polymorphism (SNP) rsPS in 16,061 Swedish and 2770 Finnish subjects followed for a median of 23.5 years. The rsPS alone (adjusted for age and sex) predicted diabetes incidence with an AUROC of 0.62, but this compared poorly to a mix of baseline clinical factors (age, sex, a family history of diabetes, BMI, blood pressure, triglycerides, fasting plasma glucose) that claimed an AUROC of 0.74. Adding the rsPS to these clinical factors had only a modest impact on performance, pushing the AUROC to 0.75. Adding genetic factors to clinical factors reclassified 9% and 20% of subjects from the Swedish and Finnish studies respectively, to a higher risk category.

In a similar study, Meigs *et al.* (31) assessed an 18-SNP rsPS in 2377 participants of the Framingham Offspring Study during 28 years of follow-up. The AUROC for incident diabetes with the rsPS alone (adjusted for age and sex) was 0.58 whereas an enhanced clinical model incorporating age, sex, family history, BMI, fasting glucose, systolic blood pressure, high-density lipoprotein cholesterol, and triglyceride levels reached 0.90. Adding genetic data to such a well-performing clinical model left the AUROC unchanged and resulted in risk reclassification of, at most, 4% of the subjects. A study of the power of an 18-SNP rsPS to capture T2D case-control status in 4907 participants from Dundee (Scotland) reached similar conclusions: the AUROC for genetics alone was 0.60, whereas the equivalent metric for age, BMI, and sex was a vastly superior 0.78, with only a slight increment (to 0.80) for the combined analysis (32).

In the decade since, waves of successively larger T2D GWAS efforts have brought the number of significant loci discovered into the hundreds. Concomitant improvements in the performance of rsPS have been more modest. An updated analysis of a 62-SNP rsPS performed in the Framingham Offspring Study (41) generated a much-improved AUROC for T2D prediction (combined with age and sex) of 0.72, but as before, the addition of genetic information provided negligible improvement in performance over the equivalent clinical predictor (AUROC for clinical factors alone, 0.90; for the combined clinical and genetic score, 0.91). Predictive performance in a second prospective study [Coronary Artery Risk Development In young Adults (CARDIA)] was uniformally worse, particularly in participants of African descent (41).

The studies so far described used rsPS, restricting the score to variants that demonstrated genome-wide significant associations. In principle, the expansion of the score to accommodate additional information from subthreshold variants should improve performance. Indeed, in a model-based analysis of predictive performance for T2D and other traits that were extrapolated from estimates of GWAS effect-size distribution and heritability (as available in 2012), Chatterjee *et al.* (42) deduced that a 10-fold increase in effective GWAS sample size for T2D (to ∼220,000) would result in a boost in rsPS performance from 0.57 to 0.74, with a further increment in performance to 0.79 if a more liberal cutoff for variant inclusion was adopted.

**Figure 2.**
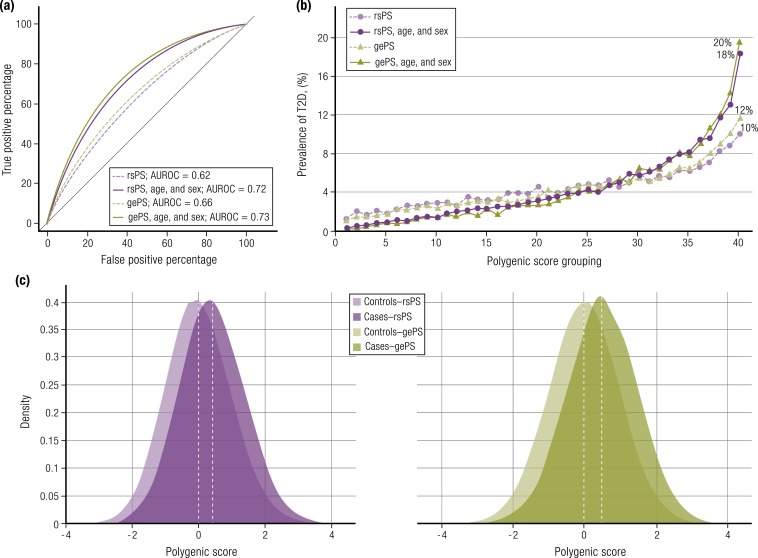
Comparison of rsPS and gePS for T2D using data from Mahajan *et al.* (9). rsPS and gePS were generated using a T2D GWAS meta-analysis of 455,313 European individuals and used to predict incident T2D in 13,480 cases and 311,390 controls from the UK Biobank. (a) AUROC curves for models predicting incident T2D: each model was adjusted for genotyping array and the first six principal components of ancestry. (b) Prevalence of T2D according to 40 groups binned according to the polygenic scores, with each grouping representing 2.5% of the population. (c) Distribution of rsPS and gePS in the cases and controls. The *x*-axis represents polygenic score, with values scaled to a mean of 0 and standard deviation of 1. Both rsPS and gePS in UK Biobank individuals is normally distributed with a shift toward the right, observed for T2D cases.

Sample sizes on that kind of scale are now within reach for T2D but, as yet, those theoretical estimates have not been realized, most likely because some of the assumptions of the model, such as heritability, were overestimated. In analyses that update those reported in the original manuscript (9), we include here a gePS generated by Mahajan *et al.* (9) from a T2D GWAS meta-analysis of almost 460,000 European individuals (effective sample size ∼158,000) that captured ∼20% of the variance in individual predisposition to T2D (about half the total estimated heritability). An optimized gePS comprising 171,249 variants was constructed using 5639 cases and 112,307 controls from the UK Biobank and then used to predict T2D case-control status (as a proxy for prospective T2D incidence) in a separate set of 13,480 cases and 311,390 controls, also from the UK Biobank. The AUROC generated was 0.66 without adjustment for age and sex, increasing to 0.73 when age and sex were added [Table 1 (9, 13, 43, 44); Fig. 2]. Khera *et al.* (13) used an analogous approach with a deeper gePS of almost 7 million variants that, after factoring in age and sex, generated a similar AUROC (0.72). Both studies found that individuals from the UK Biobank (who were aged between 40 and 69 years at recruitment and tended to be relatively healthy) in the top 2.5% to 5% of the gePS distribution were at an approximately threefold increased risk (case-control prevalence of ∼11%) compared with the mean of the rest of the sample and at an almost 10-fold increased risk compared with the bottom 2.5% (prevalence of ∼1%) (9, 13). The former OR could be expanded to ≥5.0 in individuals with the very highest gePS, although this high-risk group constituted only the ∼150 individuals in the 0.05% extreme of the distribution (13).

**Table 1.** Comparison of Three Published Global, Extended Polygenic Scores for T2D

		Study		
		Khera *et al.*, 2018 (13)	Mahajan *et al.*, 2018 (9)	23andMe (43)
Discovery GWAS	Number of cases	26,676	55,005	80,792
	Number of controls	132,532	400,308	1,479,116
	Reference	Scott *et al.*, 2017 (44)	Mahajan *et al.*, 2018 (9)*^a^*	Multhaup *et al.*, 2019 (43)
Optimization data set	Methods	LDpred	Pruning and thresholding	Predetermined cutoffs
	Number of cases	2785	5639	48,028
	Number of controls	120,280	112,307	893,692
	*P* value threshold	—	0.1	1 × 10^−5^
	LD pruning threshold	—	*r* ^2^ > 0.6	50-kb window
	Tuning parameter	*ρ* = 0.01	—	—
	Polymorphisms in risk score	6,917,436	171,249	1244
	Reference	UK Biobank	UK Biobank	23andMe*^b^*
Testing data set	Number of cases	5853	13,480	9008
	Number of controls	288,978	311,390	167,622
	Reference	UK Biobank	UK Biobank	23andMe
AUROC in testing data set (Europeans)	Not adjusted for age and sex*^c^*	0.64*^d^*	0.66	0.65
	Adjusted for age and sex	0.73	0.73	—
	OR of top 5% bin vs remainder population	2.75	2.75 without age and sex adjustment	2.76*^d^*
			4.52 with age and sex adjustment	

For the LDpred algorithm, the tuning parameter *ρ* reflects the proportion of polymorphisms assumed to be causal for the disease. For the pruning and thresholding strategy, *r*^2^ reflects the degree of independence from other variants in the linkage disequilibrium, and the *P* value reflects the *P* value threshold used for selecting variants from the discovery GWAS.

Abbreviation: LD, linkage disequilibrium.

^a^ Discovery GWAS from Mahajan *et al*., 2018 (9) after removing UK Biobank samples. Note the difference in testing data set sample size from the published results in Mahajan *et al*., 2018 (9). Results presented here are based on reanalysis of data after splitting UK Biobank samples into optimization and testing sets.

^b^ Subset of GWAS samples.

^c^ Logistic model adjusted for other technical covariates such as principal components.

^d^ Obtained through private communication with authors.

One interesting feature to emerge from the reanalysis of the T2D polygenic scores shown in Fig. 2 [based on the data from (9)] is the limited increment in performance seen between the rsPS (which was based on 199 genome-wide significant SNPs) and the gePS (built from ∼170,000 SNPs). A second observation is the reassuring concordance in the estimates of predictive performance obtained in these two studies (9, 13), despite differences in the methods, although it is worth noting substantial overlap in the data sets used for training and testing (Table 1). Furthermore, these risk estimates are almost identical to those generated by the direct-to-consumer company 23andMe from their data set of ∼1 million individuals (mean age <50 years) (43). 23andMe has recently started sharing results from their 1244-SNP T2D gePS with their customers, with a recommendation that those deemed at high risk consider lifestyle interventions to mitigate that risk. A T2D risk score generated by Genomics PLC had similar performance (45).

### Predicting T1D onset

Although clinical management strategies exist to prevent the development of T2D in those determined to be at high risk (39, 40), there is currently no known effective strategy to prevent T1D. Nevertheless, genetic profiling could have value in defining individuals at the highest future risk of T1D for enhanced surveillance or inclusion in trials of early immunologic interventions. In turn, when those trials are successful, could prove instrumental in stratifying those most likely to benefit from new preventative approaches.

Similar to T2D, T1D has a substantial heritable component, estimated to be between 65% and 88% (46–48). Genetic variation in the HLA region on chromosome 6p21 accounts for ∼50% of that heritability (49). The DR and DQ loci confer the strongest association with ORs as high as 16 for the DR4-DQ8/DR3-DQ2 genotype (50). Subsequent GWAS have identified >50 non-HLA genetic loci contributing to T1D risk, including SNPs near the *INS*, *PTPN22*, and *CTLA4* genes with substantial impact on T1D risk (10, 51–53).

During the last 15 years, genetic prediction for T1D has evolved from the use of HLA alleles alone (54) to incorporation of >40 non-HLA variants (55–58). Two rsPS for T1D developed independently by Winkler *et al.* (56) and Oram *et al.* (57) were recently merged into a single rsPS including 41 HLA and non-HLA SNPs (59). This 41-SNP rsPS was deployed within the T1D in the Environmental Determinants of Diabetes in the Young (TEDDY) study, which followed several thousand children with high T1D–risk HLA genotypes from birth, using the development of islet autoantibodies and diabetes as outcomes indicating disease progression. The 41-SNP T1D rsPS successfully stratified risk: children with a score >14.4 had 11.0% risk of developing multiple islet autoantibodies by age 6 years and 7.6% risk of diabetes by age 10 years, compared with those with scores below this who had rates of 4.1% and 2.7%, respectively (59).

By leveraging advances in density of SNP arrays as well as larger reference panels, the most recently updated T1D rsPS includes 67 SNPs and accounts for interactions between 18 HLA DR-DQ combinations (60). When applied to the UK Biobank, this enhanced T1D rsPS significantly outperformed previous scores, identifying individuals with T1D with an AUROC of 0.92. These figures are close to the maximum performance figures predicted for T1D, based on the modeling analyses described earlier in the context of T2D (42).

### Refining the diagnosis of major diabetes subtypes

As well as predicting future disease risk, polygenic scores are emerging as powerful tools to support diagnosis of major diabetes subtypes. Determining whether a particular patient has T1D, T2D, or one of the other specified forms of diabetes is not always straightforward. A clinical diagnosis of T1D can often, but not always, be substantiated by the presence of one or more islet autoantibodies (GAD, IA2, IAA, ZnT8), as these are found in >90% of newly diagnosed patients (61). However, these antibodies are not always measured in clinical practice, and they do not provide perfect determination of T1D diagnosis due to a combination of (i) background presence in some individuals without T1D, (ii) lower rates of positivity for T1D individuals diagnosed in adulthood, and (iii) waning titers over time from initial diagnosis (57). The measurement of C-peptide levels in plasma or urine can also help distinguish T1D from other forms of diabetes, but use of this test is not routine, not least because it has reduced value at the time of diagnosis (where it can be suppressed even in T2D or monogenic diabetes) or during the “honeymoon period” of T1D, given residual *β*-cell function in the early years following presentation (62). The consequence is relatively high rates of both underdiagnosis and overdiagnosis of T1D when trying to differentiate it from both T2D and less common forms of diabetes, such as MODY (63). The stable nature of a polygenic score, unchanged throughout life, offers a useful tool to aid in diagnostic characterization of individuals with established diabetes.

An early application of the initial T1D rsPS developed by Oram *et al.* (57) was in discriminating between T1D and T2D. The authors applied both a 69-SNP T2D rsPS and a 30-SNP T1D rsPS to a sample of well-defined cases of T1D and T2D from the Wellcome Trust Case Control Consortium GWAS (64). They found that the T1D rsPS was highly discriminative (AUROC of 0.88), whereas the T2D EPS was less so (AUROC of 0.64), and that combining the two offered little improvement beyond the T1D score alone (AUROC of 0.89) (57).

Application of the 30-SNP T1D rsPS alone to a cohort of 223 adults, aged between 20 and 40 years diagnosed with diabetes at least 3 years previously, predicted progression to insulin deficiency (AUROC of 0.87) and offered information additional to that provided by antibody status (57). In 8608 individuals with a clinical diagnosis of T2D after 35 years of age, treated without insulin for at least 6 months following diagnosis, the same T1D rsPS predicted progression to insulin use at 5 years, but only in the small subset of GAD antibody-positive participants: the probability of insulin use ranged from 17.6% in those in the lowest tertile of T1D risk to 47.9% in the highest (65).

T1D polygenic scores have also provided a clearer sense of the extent of T1D prevalence across the age spectrum. Using a 29-SNP T1D rsPS, Thomas *et al.* (66) demonstrated that, among individuals participating in the UK Biobank, 42% of genetically defined T1D was observed in those diagnosed with diabetes between 31 and 60 years of age, pointing to a far higher proportion of overall T1D presenting in adulthood than is commonly appreciated. It can be challenging to detect these individuals clinically because, in this age range, they represent only a small minority (∼4%) of patients with any form of diabetes. Compared with those with T2D, individuals with T1D defined on the basis of a high T1D rsPS had lower BMI, were more likely to use insulin in the first year of diagnosis, and were at higher risk of diabetic ketoacidosis (66).

T1D polygenic scores have also shown utility in discriminating early-onset T1D from monogenic forms of diabetes, including MODY (67), neonatal diabetes (67), and monogenic autoimmune diabetes (68), which typically present during childhood. In these settings, T1D scores can prioritize patients who are most likely to benefit from sequence-based testing for rare causal variants, and support correct interpretation of novel variants of uncertain functional significance that emerge from such sequencing. Prioritization of patients in this way is important both for providing a cost-effective strategy to increase diagnosis rates for known forms of monogenic diabetes and for facilitating new gene discovery by reducing study subject heterogeneity. A related application of polygenic scores may be to explain some of the variable presentation of monogenic forms of diabetes, with respect to age of diagnosis for example (69). The same variant within the *HNF1A* gene may segregate with early-onset diabetes in some pedigrees, but it also may be observed in individuals who retain normal glucose tolerance into late adulthood and beyond (70). Studying 410 individuals from 203 *HNF1A*-MODY families, Lango Allen *et al.* (71) found that a 15-SNP T2D rsPS was significantly associated with earlier age of diabetes diagnosis, with each additional risk allele accelerating diagnosis by ∼4 months.

### Clinical application of predictive scores

These data provide a sound basis for the use of polygenic scores to support discrimination of major diabetes subtypes and lend credence to their wider clinical value. Given analogous applications of the polygenic score approach for other multifactorial disease traits (13, 72, 73), these findings have collectively bolstered excitement about their potential to deliver clinical benefit across a wide range of common diseases.

One major focus of current research activity lies in exploring the value of polygenic scores to predict individuals at the highest risk of T2D so as to enable early targeting of intervention strategies. If the estimates of relative risk seen in UK Biobank participants in recent studies generalize to the population level [and the current data indicate that performance seems to be sustained throughout the age ranges studied (9, 43)], then there are likely to be >1 million individuals in the United Kingdom, who, on the basis of their polygenic score alone, have a ∼50% lifetime risk of T2D (9). With the price of whole-genome sequencing falling, and the potential to achieve near-perfect imputation by harnessing the combination of large-scale whole-genome sequencing (in a subset of the population) and dense GWAS arrays (in the rest), several countries are starting to plan for a future of universal genetic screening. The rationale is that “one-time” measurement of genome-wide genetic variation (achievable for the cost similar to that of a single outpatient appointment or a chest X-ray) would support a wide range of clinical applications throughout a person’s lifetime, including, but not limited to, the optimization of therapies (based on pharmacogenetic insights) and the prediction of future illness using polygenic scores for a range of diseases.

However, clearly multiple obstacles must be overcome before this becomes the standard of care. First, there are technical issues. The most critical among these involves ensuring that polygenic scores are appropriately calibrated to the ethnicity of the individual being tested. An rsPS or gePS generated using data solely derived from Europeans will have suboptimal ability to capture risk in individuals of non-European origin. The T2D gePS recently released by 23andMe demonstrates a marked fall-off in predictive performance in individuals of Asian and African American origin (43). In some settings, these issues with the transethnic portability of polygenic scores go beyond a simple dilution of performance: unpredictable biases and the consequences of genetic drift can result in entirely misleading results (74, 75). Recent studies have also emphasized the impact of residual population stratification effects on the performance of these scores (76, 77). RsPS are likely to be more robust to these biases than gePSs.

The second question to be addressed concerns whether a given polygenic score adds clinical value to the predictions that are possible using existing risk factors. In the case of coronary artery disease, there is evidence that a substantial proportion of those at highest polygenic risk would not have been detected using classical risk factors (13). In contrast, and as described earlier, the incremental benefit of a polygenic score over easily accessible clinical parameters seems more limited for T2D, at least when applied at older ages. In fact, the nongenetic risk factors we already collect in clinic (family history, ethnicity, BMI, fat distribution) perform quite well in predicting T2D, particularly in the near term, especially when supported by direct biomarkers of the underlying disease process such as measures of glycemia (30–32, 41). There is an intrinsic limitation to the added value of a polygenic score arising from the fact that trait heritability provides a ceiling for the performance of any purely genetic measure.

Third, there is the issue as to whether early diagnosis can be shown to result in beneficial outcomes, for example by motivating improvements in lifestyle or treatments that reduce the risk of disease. In the case of T2D, the potential for lifestyle modification and/or pharmaceutical intervention (*e.g.*, with metformin) to reduce diabetes progression is clear (39, 40), and these benefits seem to accrue irrespective of genetic risk. In the Diabetes Prevention Program, for example, lifestyle intervention was effective at reducing diabetes incidence compared with placebo even among those with the highest quartile of T2D rsPS (78). However, there is limited evidence to date that the communication of genetic risk is sufficient to motivate most individuals to undertake the kind of long-term behavioral modification required for sustained benefit (79–81). There is also some (at least theoretical) risk of harm if the communication of risk information is mishandled. This could arise through failure to use ethnically appropriate scores, or to incorporate other relevant health information. For example, an overweight person with a low T2D polygenic score may be at far greater risk of disease than the polygenic score alone would suggest. Some individuals may be liable to interpret high genetic risk in a deterministic and fatalistic way, failing to appreciate that remediation of risk through lifestyle modification is no less likely to be effective in their case.

Finally, there are questions related to implementation. Several countries (Finland, Estonia, United Kingdom, and Taiwan, among others) are expanding the clinical roll-out of genome-wide genetic data, with plans to deliver genetic profiling to the population scale through a combination of sequence- and array-based strategies. Such universal availability of genomic data would open up much wider use of polygenic scores: the costs of acquiring such data (which needs to be done only once in the life of an individual) could be amortized across multiple applications (rather than needing to be justified based on any single indication), and the marginal costs of any specific use of those data would be minimal. Having said that, any valid assessment of clinical utility needs to consider the full costs of any given application: if the consequence of the unregulated use of genetic information is to identify a large proportion of the population at high risk, there may be substantial financial and health costs to be incurred in follow-up screening, unnecessary treatment, patient stress, and the unproductive use of medical resources. A rigorous pipeline for the interpretation of these findings and their translation into evidence-based clinical interventions at the point of care will need to be created and deployed for multiple phenotypes across multiple health care systems.

## Partitioned Polygenic Risk Scores

So far, in this review, we have focused on the use of restricted (rsPS) and expanded (gePS) polygenic scores, both of which aim to capture the genetic contribution to predisposition for the major disease phenotypes conventionally used to define morbid states, such as T1D and T2D. These scores are designed to enable prediction of an individual’s risk of developing one of these forms of diabetes, or, as described above, to support differential diagnosis in those who have recently been diagnosed with diabetes. For these indications, it makes sense to combine as many risk variants as possible, irrespective of the mechanisms through which they influence that risk.

*“Access to an expanded range of large-scale quantitative trait association data…have enabled a new wave of variant clustering analyses.”*

However, these are not the only clinical questions that polygenic scores are equipped to address. Many of the most difficult problems in the clinical management of T2D, in particular, arise out of the clinical and phenotypic heterogeneity that is an obvious feature of this condition. Clinical management of someone with a diagnosis of T2D would be substantially improved if it were possible to sense how fast their diabetes is likely to progress, their propensity for developing macrovascular and microvascular complications, and their likely response to the range of treatments (therapeutic, surgical, and behavioral) that could be deployed to improve outcomes. Because these are questions that relate to clinical and etiological heterogeneity in those with established T2D, polygenic scores based on overall disease risk are unlikely to offer discriminatory value.

As discussed earlier, one promising route to capture elements of this clinical heterogeneity is through the use of pPS. These seek to “deconstruct” the overall (restricted or extended) polygenic score along biological axes that represent contributory etiological pathways, and thereby provide a framework upon which to map the variable response to clinical outcomes.

One way of conceptualizing these pPS is in terms of the “palette” model of diabetes predisposition, which seeks to focus attention not on T2D itself, but on the various intermediary processes that collectively contribute to T2D risk (3, 38). These include well-studied processes such as obesity, fat distribution, islet development and function, and insulin sensitivity, although there are likely to be others that are, as yet, less clearly described. Each of these processes is itself under multifactorial (genetic and nongenetic) control, and a given individual may be positioned at any point on the spectrum from “low T2D risk” to “high T2D risk” for each of these. Although the overall load of T2D risk across the set of processes is likely to be a useful measure of the overall T2D risk of an individual, the disposition of that risk across the various axes is likely to be more informative regarding disease presentation and clinical course. In accordance with the palette analogy, each of these processes can be considered to be represented by a particular base color (*e.g.*, red, blue, yellow): for any given individual, risk along each axis would be captured by the saturation of the relevant base color and his or her overall profile of T2D predisposition visualized in terms of the mix of those colors that results when they are combined.

This palette model is consistent with current understanding of the pathogenesis and the genetic architecture of T2D. During the past decade, T2D-associated variants have been shown to modulate T2D risk through diverse mechanisms: some increase T2D risk through an impact on obesity (*e.g.*, *FTO*), others reduce insulin sensitivity (*e.g.*, *PPARG*, *IRS1*), whereas others compromise insulin secretion, either through direct effects on islet function (*e.g.*, *KCNJ11*) or development (*e.g.*, *HNF1A*) or indirectly through impact on incretin signaling (*e.g.*, *GLP1R*) (82). The various classes of T2D therapeutics operate through the same range of mechanisms to reverse the diabetic phenotype or control its glycemic consequences. The weight of evidence indicating that the genetic contribution to T2D predisposition mostly arises from common variants of limited individual effect (11, 12) emphasizes the need to think in terms of a gradation of polygenic risk across individuals, rather than a classification based around rigid, discrete subtypes (3). As well as providing a framework for capturing the mechanistic basis of T2D heterogeneity, this model also offers an approach to understanding how an individual’s particular genetic profile contributes to their progression from normal metabolic health toward the diabetic state.

In 2010, Voight *et al.* (19) were the first to demonstrate that patterns of genetic association across diabetes-related quantitative traits could be used to annotate T2D-risk loci with respect to their physiological impact, analyses that highlighted the predominant role played by variants influencing insulin secretion. This approach was further developed by Dimas *et al.* (83) to perform a systematic analysis of the relationships between 36 T2D-risk alleles and a range of glycemic measures, including indices of insulin secretion and insulin resistance gathered in nondiabetic individuals. Scott *et al.* (44) extended this approach to a larger set of 93 T2D-risk alleles and included BMI and lipid measures in their clustering in addition to glycemic traits. Three main patterns of multitrait association emerged from this analysis, two of them reflecting defects in insulin secretion and insulin action, respectively, and a third characterized by obesity and dyslipidemia. One major limitation of the unsupervised hierarchical “hard” clustering approach used in these studies (44, 83) is that it requires each variant to be assigned to a single cluster, based on the questionable assumption that each variant can only be involved in one pathophysiological pathway.

Access to an expanded range of large-scale quantitative trait association data [from large-scale GWAS efforts within global consortia such as GIANT (anthropometric traits), MAGIC (continuous glycemic traits), and GLGC (lipids)] plus advancements in clustering algorithms have enabled a new wave of variant clustering analyses (20, 38). These described efforts to aggregate GWAS data from more diverse sets of T2D-related quantitative traits and used more sophisticated “soft” clustering techniques (84, 85) to pick out clusters of T2D-associated variants with similar patterns of impact across the suite of phenotypes. These soft clustering approaches explicitly allow for the possibility that a variant influences more than one process. Mahajan *et al.* (20) deployed a c-means clustering approach across GWAS data from 10 T2D-related quantitative traits for a set of 94 T2D association signals that emerged from a T2D GWAS of ∼450,000 individuals, identifying six variant clusters (based on a threshold of 80% for cluster membership). Udler *et al.* (38) used a complementary soft clustering approach—Bayesian nonnegative matrix factorization—to a partly overlapping set of 94 T2D-risk variants, gathering GWAS data from 47 diabetes-related traits, and identifying five clusters. Reassuringly, despite these differences, the clusters identified by both were broadly similar [Table 2 (20, 38)].

**Table 2.** Partitioned Polygenic Score Clusters Capturing Etiological Heterogeneity in T2D

Physiological Impact		Phenotypic Features	Cluster Name		
			Udler *et al.*, 2018 (38)	Mahajan *et al.*, 2018 (20)	Examples of T2D Loci
Adverse impact on *β*-cell function	High proinsulin	Low fasting insulin (+ high proinsulin)	*β*-Cell	Insulin secretion 1	*ABO*, *ADCY5*, *HNF1A*, *HNF1B*, *MTNR1B*, *SLC30A8*, *TCF7L2*
	Low proinsulin	Low fasting insulin (+ low proinsulin)	Proinsulin	Insulin secretion 2	*IGF2BP2*, *CENTD2/ARAP1*, *CCND2*
Reduced insulin sensitivity	Mediation with fat distribution	High fasting insulin + low BMI + low WC + high TG	Lipodystrophy	Insulin action	*MACF1*, *GRB14*, *IRS1*, *PPARG*, *ANKRD55*, *KLF14*, *LPL*, *CMIP*
	Mediation via obesity	High fasting insulin + high BMI + high WC	Obesity	Adiposity	*NRXN3*, *FTO*, *MC4R*
	Mediation via lipid metabolism	Low TG	Liver/lipid	Dyslipidemia	*GCKR*, *TM6SF2*
Undetermined		No striking phenotype association	No assignment	Mixed features	*BCL11A*, *TLE1*, *PLEKHA1*, *HMGA2*, *MTMR3*

Comparison of pPS clusters identified by Mahajan *et al.* (20) and Udler *et al.* (38).

Abbreviations: TG, triglyceride; WHR, waist/hip ratio.

The variants within each of the genetic clusters can be used to generate “partitioned” polygenic scores that capture the genetic contribution to each intermediary process. Each of these clusters (and the pPS generated therefrom) can be assigned mechanistic labels based on the observed patterns of GWAS effects; for example, a cluster that features T2D-risk alleles most clearly associated with decreased fasting insulin can, on the basis of known pathophysiological relationships, be attributed to reduced insulin secretion. On this basis, two of the clusters were associated with an adverse impact on *β*-cell function, three were characterized by insulin sensitivity (differing with respect to their relationship to obesity, fat distribution, and lipid metabolism), and a sixth cluster [designated only in the Mahajan *et al.* (20) paper] had less clearcut phenotypic features (20, 38) (Table 2).

The T2D-risk variants assigned to the three insulin sensitivity clusters displayed the most obvious overlap across the two approaches. Variants near *FTO*, *MC4R*, and *NRXN3*, all loci known to have substantial impact on variation in BMI, mapped to a cluster of T2D-risk variants thereby assumed to be driven primarily by obesity. Variants at *IRS1*, *PPARG*, and *KLF14* implicated in effects on adipocyte differentiation and body fat distribution were colocated to a cluster of T2D-risk variants featuring lipodystrophy-like effects on insulin sensitivity, partly overlapping with the set of “favorable adiposity” loci identified by others (34–36). Finally, variants at *GCKR* and *TM6SF2*, known for their profound impact on ectopic fat accumulation in liver and altered circulating lipid levels (86, 87), were members of a cluster that seems to be driven by alterations in hepatic metabolism.

Although there was broad agreement concerning the variants deemed to influence *β*-cell function, disposition across the pair of *β*-cell clusters was less consistent, particularly for variants with less dramatic effects on the continuous glycemic traits that distinguished them. T2D-risk variants at *SLC30A8*, *TCF7L2*, *ADCY5, HNF1A,* and *MTNR1B* consistently mapped to a cluster characterized by an association between T2D risk, reduced insulin levels, but elevated proinsulin levels, whereas those at *ARAP1*, *IGFBP2*, *DGKB*, and *CCND2* combined T2D risk and reduced *β*-cell function with reduced proinsulin levels. Some of the variation in the assignment of other variants across these two clusters reflects differences in the traits included in the respective analyses, compounded by substantial differences in the size of the GWAS data sets available across traits (which has an impact on discriminatory power). Nevertheless, the replicated subdivision of *β*-cell function variants into two clusters distinguished by the direction of the association to proinsulin speaks to two distinctive mechanisms whereby T2D-associated variation results in *β*-cell dysfunction (88).

Despite some of the differences in the assignment of individual variants across clusters, the mechanistic basis of these clusters appears robust, mapping as it does to current understanding concerning the major pathophysiological processes influencing T2D development. Allocation of variants to these physiologically defined clusters is also broadly supported by orthogonal analyses of tissue-specific patterns of chromatin accessibility, histone modification, and transcriptional regulation. The various subsets of T2D-risk variants identified by clustering of GWAS data demonstrate clear evidence of genome-wide enrichment with respect to tissue-specific active enhancers and promoters (9, 38, 44, 89–91), *cis*-eQTL signals (90, 92), and enhanced connectivity in tissue-specific protein–protein interaction networks (93). As anticipated, these link variants in the insulin secretion clusters to altered transcriptional regulation in the islet, and those in insulin action clusters to events in liver, fat, and muscle.

Beyond the ability of these efforts to identify disease pathways, a critical question in terms of clinical translation is whether the pPS generated from these clusters show associations with clinically relevant outcomes: early results are encouraging. For example, differential cluster associations have been observed for coronary artery disease, stroke, and the renal complications of diabetes (38, 94, 95), each emphasizing enhanced risk associated with T2D predisposition mediated through insulin resistance. In the case of macrovascular disease, of course, this is likely to reflect the pleiotropic impact of these variants on nonglycemic risk factors such as lipids. A specific role for pPS-captured defects in insulin secretion and altered gut microbiome has also been reported: those microbiome changes include an effect on butyrate-producing pathways shown to play a causal role with respect to diabetic and obesity phenotypes (8).

These findings support the notion that although, by definition, all cluster-defined pPS associate with T2D risk, differential effects can be detected with respect to aspects of mechanism, phenotype, and clinical outcomes. However, further effort is needed to validate and extend these findings, and to define the contribution that these can make to the delivery of more personalized management in diabetes. So far, clustering analyses have been restricted to a subset of the most robust genome-wide significant T2D-associated variants, primarily those discovered in Europeans, and for which association statistics are available across multiple related traits. More complete analyses (delving deeper into the list of T2D-associated variants, and embracing a wider range of traits) capable of generating more powerful pPS will become possible as GWAS efforts for those other traits scale up. Inclusion of additional phenotypes should provide more granular clustering, attributing mechanisms to variants that currently show only weak phenotypic features, and bringing to light new pathways involved in T2D development. Integration with tissue- and cell type–specific regulatory annotation maps will continue to support mechanistic inference (38, 44). Greater access to association data on T2D and other traits from non-European ethnicities will enable broader exploration of ethnic-specific variants and the heterogeneity of clinical presentation and course across major ethnic groups. As confidence grows in the mechanistic basis of these variant clusters, it will become possible to use trait-specific GWAS data to “build out” cognate pPS and generate more powerful genetic instruments. For example, the pPS formed from the handful of genome-wide significant T2D variants in the “obesity” cluster could be superseded by using a polygenic score constructed from the BMI GWAS efforts themselves, and a pPS capturing islet autoimmunity generated from existing polygenic scores for T1D.

For diseases such as T2D, the characterization of clinical phenotype using genetic measures alone is constrained by the fact that individual variation within each of the endophenotypic axes is also influenced by nongenetic factors. Diagnostic and predictive accuracy would be much improved, and the ability to track an individual’s journey from health to disease much enhanced, if the genetic contribution to phenotypic variation (as captured by the pPS) can be integrated with robust longitudinal measures of relevant features of the external environment (*e.g.*, related to diet and physical activity) and internal milieu (*e.g.*, metabolic memory and microbiome). Integration of this “predictive” information with evolving measures of the individual’s clinical state would add another dimension. In the context of T2D, the latter would involve capturing anthropometric data, and the glycemic and metabolic states, forming an integrated profile of that individual that can be tracked over time.

It would be particularly valuable in this regard to develop process-specific biomarkers that provide clinical readouts for each of the endophenotypic axes that corresponds to a particular pPS. The best illustration of this concept is the use of low-density lipoprotein cholesterol as an integrated biomarker for that component of cardiovascular risk attributable to genetic and environmental influences on lipoprotein metabolism. The growing availability of large, publicly available metabolomic and proteomic data sets makes it possible to use pPS as instruments to identify biomarkers correlated to pPS-defined risk as candidates for further prospective testing (96, 97).

A key focus of ongoing research relates to understanding how these pPSs might be deployed in clinical practice. One interesting possibility is that pPS profiling will allow identification of subsets of individuals whose diabetes is mostly attributable to defects in a single process. In the analysis by Udler *et al.* (38), one third of individuals fell within the top decile of T2D risk for at least one cluster and, of these, 75% were not placed at the top decile of any other cluster. These individuals would be obvious recruits for the testing of targeted interventions. An alternative, possibly complementary, approach would make use of the full range of scores for a given individual to assign risk and optimize management. In either case, much will depend on the extent to which these various ways of representing etiological heterogeneity (with or without additional environmental and clinical state information) can be shown to optimize clinical management (*e.g.*, the selection of therapeutic agents).

One important corollary is that, by conceptualizing a disease such as T2D as arising from the coming together of diverse, largely orthogonal underlying processes, these models question some of the tacit concepts underlying precision medicine. One of these is the notion that characterization of the specific defect contributing to an individual’s disease invites therapeutic approaches that are designed to specifically correct it. This model has proven effective in monogenic diabetes—where one molecular defect is largely responsible for the phenotype—but it is less clear this can be implemented in polygenic disease. In people in whom the disease is caused by multiple processes, it will be unlikely that modulating a single pathway will be sufficient to correct metabolic derangements, whereas in those in whom the contributions of specific genetic defects are modest, equivalent reductions in disease risk and progression may be possible through interventions that boost the performance of other processes contributing to overall T2D risk, even those that are already performing at healthy levels. Indeed, because the effects of common variants on the hyperglycemic phenotype are modest, current T2D drugs that target specific pathways (*e.g.*, sulfonylureas and thiazolidinediones) appear to be effective in both carriers and noncarriers of T2D-associated alleles in the respective target-encoding genes (98, 99). Nevertheless, it is possible that some individuals will be identified whose pathophysiology is predominantly driven by one process, and in whom the monogenic paradigm of a drug targeting that very process could be applied effectively. Whether, and in whom, such approaches may prove successful will require the conduct of appropriately designed precision clinical trials.

These pPS approaches to analyzing phenotypic heterogeneity, which build out from genetic risk, offer a complementary perspective to the results emerging from the analysis of real-world data (100, 101). These real-world methods have focused on efforts to classify T2D into distinct subtypes, analogous to the categorization of monogenic forms of disease. Such an objective, if successful, would offer clinical expediency.

However, these efforts to sift individuals into discrete subtypes of disease would appear to run counter to the evidence that points to a complex, graded, architecture of risk, one that is consistent with a multifactorial etiology, composed of genetic predisposition dominated by multiple common variants of modest effect, and pervasive exposures contributing to risk. In one recent study, Ahlqvist *et al.* (101) used basic clinical information from patients with newly diagnosed adult-onset diabetes to define five subtypes of late-onset diabetes: an autoimmune form (covering T1D and other related clinical entities), two severe forms (one dominated by insulin deficiency, the other by insulin resistance), and two milder forms (termed “obesity-related“ and “age-related” diabetes). Whereas the genetic clusters that form the basis of pPS are defined at the level of the variants, these clinical subtypes are defined at the level of the individual and based on biomarkers and clinical data gathered at a specific point in the progression of an individual from health to disease. The latter is likely to limit their relevance to those who have not yet developed disease, and/or those who are on treatment.

It is worth emphasizing the different, but complementary, nature of these two approaches: the partitioned risk approach involves first clustering genetic signals by mechanism to derive pPS, and then exploring how the quantitative pPS perform across individuals. In contrast, the phenotypic clustering approach attempts to hard cluster individuals on the basis of their physiology. Further work is required to understand how these two approaches to capturing clinical heterogeneity relate to each other, and to objective measures of clinical utility. One of the fundamental issues, which pervades diverse aspects of precision medicine, relates to the relative merits of retaining as much quantitative information on an individual as possible until the point when a substantive (typically binary) clinical decision needs to be made, as opposed to early diagnostic categorization of the individual in a way that bases subsequent clinical decision-making on the optimized outcomes of the group to which they have been assigned. Although further investigation is needed, a recent analysis by Dennis *et al.* (102) in the A Diabetes Outcome Progression Trial (ADOPT) and Rosiglitazone Evaluated for Cardiac Outcomes and Regulation of glycemia in Diabetes (RECORD) clinical trials indicated that the former approach—considering phenotypic traits as continuous measures—provided better predictive value of treatment response than did an approach that binned individuals using the phenotypic clustering approach of Ahlqvist *et al.* (101).

## Summary and Further Discussion

After many years of frustration at the slow progress that had been made in the translation of recent discoveries in human genetics—notably the many risk variants for common, multifactorial forms of diabetes identified through GWAS and sequencing—there is now growing optimism that the use of polygenic scores will offer substantial clinical benefit and contribute to efforts to forestall the growing morbidity and mortality associated with these conditions. Some early clinical applications have emerged, mostly related to positive identification of those who have developed, or are at the highest imminent risk of developing, T1D (57, 65–68).

*“…genetics represents only one contributor to individual disease risk and profile….”*

It is inevitable that clinical applications of the polygenic score approach will roll out at a different pace across disease conditions, with a focus on different clinical questions, dictated by the additional clinical benefit that they provide, and the extent of the unmet clinical need. One size certainly does not fit all, and the relative merits of the different types of polygenic score described in this review (gePS, rsPS, pPS) will differ according to the specific clinical situation. It also remains to be determined how or whether pPS and phenotypic trait clustering will impact clinical care and be deployed in practice.

Recent developments in relationship to the potential clinical use of polygenic scores have led to heated debate between those who are enthusiastic about the potential and those who are of the view that the clinical value of human genetics discovery has been consistently hyped and who feel that polygenic scores represent just the latest chapter in that story of scientific overselling (103, 104). As in other similar situations, the outcome of this debate will become clearer as theoretical and basic knowledge develops and the collection of real-world data expands. However, it is already possible to identify a series of obstacles that need to be overcome before the full potential of this approach can be realized.

Most critical is the need to ensure that the benefits of accurate, robust polygenic score determination are equally available to all. As others have pointed out, most GWAS and sequence data have been derived from the European-descent individuals who live in the developed nations of Europe and North America, and the polygenic scores generated from these data perform best when applied to the same populations (74, 75). There is a critical need to generate equivalent data and polygenic scores in other populations, to explore and characterize the extent to which transethnic portability of polygenic scores can be tolerated, and to define strategies for their deployment in special situations such as recently admixed and isolate populations. Concerns about the impact of population stratification and the limits of transethnic portability provide arguments for the use of rsPS over gePS (74–77). This may be particularly true for T1D and T2D given the limited increment in performance available with more extended scores.

Wider recognition needs to be given that, for multifactorial traits with an appreciable nongenetic component, a wholly genetic explanation of disease prediction and state will never provide a perfect clinical instrument. In some settings, the information from genetics may simply recapitulate measures already available from other risk factors. The clinical use of cholesterol measures as a biomarker for coronary artery disease risk provides an illustration of this point, reflecting the benefits it offers as an integrator of both genetic and environmental risk. At the same time, some of those who are less enthusiastic about the clinical value of polygenic scores often fail to acknowledge that many established clinical tools (*e.g.*, the use of BMI to predict T2D risk, or the use of islet cell antibodies for the differential diagnosis of T1D in late-onset diabetes) are likely to have performance metrics that limit their discriminative power. As the costs associated with the generation and interpretation of individual genomic information decline, there will be a growing roster of clinical applications where polygenic scores can add value.

There is clearly a need to develop novel approaches to establish the clinical validity and utility of polygenic scores in medical practice that take account not just of the marginal cost of acquiring the data, but the full costs of implementation. Randomized clinical trials are unlikely to be the answer here, not least because the dynamic nature of the underpinning genetic databases means that polygenic scores are likely to evolve, rapidly rendering redundant any precise quantification of cost and benefit based around a historical set of scores (105). There will need to be concomitant efforts to document the provenance, content, and performance of polygenic scores using standardized metrics and conventions that do not currently exist.

There will need to be education of citizens and professionals to appreciate the benefits and limitations of polygenic scores (106). It should be clear that genetics represents only one contributor to individual disease risk and profile, that genetically defined risk should not, for multifactorial traits at least, be considered deterministic, and that most of the evidence indicates that behavioral modifications are just as likely to succeed (and in fact to be even more beneficial) in those at highest genetic risk (78). The ease with which polygenic score information can be integrated with conventional approaches to risk profiling that are already widely used in clinical practice (*e.g.*, to estimate future risk of coronary artery disease) should facilitate widespread introduction and minimize the need for the health care professionals involved to develop an intimate knowledge of human genetics. Clearly, any clinical application of genetic data will need to address issues related to privacy and informed consent (107).

At the heart of precision medicine is the notion that an improved specification of disease risk or subtype will allow better targeted interventions to prevent or treat disease. Such efforts must compete for resources with population-based interventions that seek to achieve the same ends through nontargeted means (108). In many existing clinical settings (*e.g.* related to reducing rates of cardiovascular disease, melanoma or breast cancer), these two strategies are seen to be complementary and are pursued in parallel. The development of polygenic score-based approaches to support targeting of high-risk individuals will not alter these assessments. As now, the balance of effort between targeted and nontargeted approaches to the reduction of disease and disability will, for any clinical indication, continue to be dependent on the relative impact, cost, acceptability and sustainability of these complementary strategies.

Appendix A: **Polygenic Score Terminology**

1. Restricted-to-significant polygenic scores (rsPSs): scores composed of variants at the extreme of a statistical distribution, most usually those that pass the genome-wide significant threshold for the trait concerned.

2. Global extended polygenic scores (gePSs): scores generated from a deeper set of variants generated from genome-wide analyses, typically involving large numbers of subthreshold significant variants.

3. Partitioned or process-specific polygenic scores (pPSs): scores composed of variants grouped according to some common biological process (*e.g.*, association with a related endophenotype, tissue expression of related genes, chromatin state).

## Data Availability

All data generated or analyzed during this study are included in this published article or in the data repositories listed in References.

## Abbreviations:

AUROC: area under the receiver operator characteristic
BMI: body mass index
gePS: global extended polygenic score
GWAS: genome-wide association study
MODY: maturity onset diabetes of the young
pPS: partitioned (or process-specific) polygenic score
ROC: receiver operator characteristic
rsPS: restricted-to-significant polygenic score
SNP: single-nucleotide polymorphism
T1D: type 1 diabetes
T2D: type 2 diabetes
